# Nonsentinel Axillary Lymph Node Status in Clinically Node-Negative Early Breast Cancer After Primary Systemic Therapy and Positive Sentinel Lymph Node: A Predictive Model Proposal

**DOI:** 10.1245/s10434-023-13231-x

**Published:** 2023-02-21

**Authors:** Isaac Cebrecos, Eduard Mension, Inmaculada Alonso, Helena Castillo, Esther Sanfeliu, Sergi Vidal-Sicart, Sergi Ganau, Maria Vidal, Francesco Schettini

**Affiliations:** 1 Clinic Institute of Gynecology, Obstetrics and Neonatology, Hospital Clinic of Barcelona, Barcelona, Spain; 2 Faculty of Medicine, University of Barcelona, Barcelona, Spain; 3 Medical Oncology Department, Hospital Clinic of Barcelona, Barcelona, Spain; 4 Translational Genomics and Targeted Therapies in Solid Tumors Group, August Pi i Sunyer Biomedical Research Institute (IDIBAPS), Barcelona, Spain; 5 Department of Pathology, Biomedical Diagnostic Center, Hospital Clinic of Barcelona, Barcelona, Spain; 6 Department of Nuclear Medicine, Diagnosis Imaging Center, Hospital Clinic of Barcelona, Barcelona, Spain; 7 Department of Radiology, Diagnosis Imaging Center, Hospital Clinic of Barcelona, Barcelona, Spain

## Abstract

**Background:**

In clinically node-negative (cN0) early stage breast cancer (EBC) undergoing primary systemic treatment (PST), post-treatment positive sentinel lymph node (SLN+) directs axillary lymph node dissection (ALND), with uncertain impacts on outcomes and increased morbidities.

**Patients and Methods:**

We conducted an observational study on imaging-confirmed cN0 EBC, who underwent PST and breast surgery that resulted in SLN+ and underwent ALND. The association among baseline/postsurgical clinic–pathological factors and positive nonsentinel additional axillary lymph nodes (non-SLN+) was analyzed with logistic regression. LASSO regression (LR) identified variables to include in a predictive score of non-SLN+ (ALND-predict). The accuracy and calibration were assessed, an optimal cut-point was then identified, and in silico validation with bootstrap was undertaken.

**Results:**

Non-SLN+ were detected in 22.2% cases after ALND. Only progesterone receptor (PR) levels and macrometastatic SLN+ were independently associated to non-SLN+. LR identified PR, Ki67, and type and number of SLN+ as the most efficient covariates. The ALND-predict score was built based on their LR coefficients, showing an area under the curve (AUC) of 0.83 and an optimal cut-off of 63, with a negative predictive value (NPV) of 0.925. Continuous and dichotomic scores had a good fit (*p* = 0.876 and *p* = 1.00, respectively) and were independently associated to non-SLN+ [adjusted odds ratio (aOR): 1.06, *p* = 0.002 and aOR: 23.77, *p* < 0.001, respectively]. After 5000 bootstrap-adjusted retesting, the estimated bias-corrected and accelerated 95%CI included the aOR.

**Conclusions:**

In cN0 EBC with post-PST SLN+, non-SLN+ at ALND are infrequent (~22%) and independently associated to PR levels and macrometastatic SLN. ALND-predict multiparametric score accurately predicted absence of non-SLN involvement, identifying most patients who could be safely spared unnecessary ALND. Prospective validation is required.

**Supplementary Information:**

The online version contains supplementary material available at 10.1245/s10434-023-13231-x.

During the last decade, primary (or neoadjuvant) systemic treatment (PST) has increasingly become the first therapeutic approach for early breast cancer (EBC) patients, also when upfront resectability is possible.^1^ The main reasons lie in the higher rates of breast conserving surgery (BCS) in originally BCS-ineligible patients, the reduction in axillary lymph node dissection (ALND) rates, along with an early treatment of micrometastatic disease and the provision of tumor chemosensitivity information.^2–9^ Additionally, several new adjuvant escalated or de-escalated systemic therapeutic approaches are driven by the achievement (or not) of a pathologic complete response (pCR).^10,11^ Likewise, surgical management of the axilla has undergone an important shift toward less radicality due to the important morbidities associated with ALND. The most relevant and frequent is lymphedema, occurring in ~ 5% of patients undergoing exclusive sentinel lymph node biopsy (SLNB), and 12–25% of patients undergoing ALND,^12–14^ with incidence increasing up to six times when ≥ 15 lymph-nodes are dissected.^15^ Lymphedema is an important comorbidity, affecting different areas of physical and social functioning, bodily pain, and general and mental health.^16,17^ Since PST can reduce the ALND rate, it might lower the occurrence of lymphedema.^6,18^ At the same time, lymphedema rates after PST, especially neoadjuvant chemotherapy (NACT), have been found to be relatively high if ALND is ultimately performed, with an incidence at 3 years from surgery ranging from 25% to 58.4%, depending also on the diagnostic criterion (e.g., arm circumference increase, symptoms).^18,19^ Moreover, a recent prospective study further confirmed that NACT per se is an independent risk factor for lymphedema.^20^ For this reason, correctly identifying patients undergoing PST that should not receive postneoadjuvant ALND is an important unmet medical need.

In the case of upfront surgery, several randomized controlled trials (RCT) demonstrated that in patients with small primary tumors (cT1–2) cN0 at diagnosis, when sentinel lymph-node (SLN) are only affected by micrometastases or 1–2 macrometastases, ALND can be safely spared or replaced by radiotherapy (RT), with less comorbilities.^21–26^

In the neoadjuvant setting, in baseline cN0 cases, SLNB is preferably carried out directly after PST, to obviate the need for two separate surgeries (i.e., SLNB pre/post systemic treatment), facilitate the final definition of axillary pCR, and reduce the sentinel lymph node (SLN)-positive (+) rates without affecting the detection rate.^7^ Nevertheless, when metastases in SLN are detected in this context, evidence from the adjuvant setting cannot be easily extrapolated. Consequently, whether ALND could be safely spared in patients with initially cN0 tumors with more or less limited SLN+ after PST is currently unknown. For this reason, we sought to identify main features associated with positive nonsentinel axillary lymph nodes (non-SLN+) in cN0 EBC patients who are diagnosed with postneoadjuvant SLN+ and develop a multiparametric predictor of negative non-SLN, so to spare unnecessary ALND in this clinical setting.

## Patients and Methods

### Patients

A prospectively maintained surgical database at the Hospital Clinic of Barcelona (HCB) including all breast cancer (BC) patients treated starting from January 2013 was consulted. We extracted data from all BC patients who respected all of the following inclusion criteria:

1. Clinically T1–3 (cT1–3) or non-inflammatory cT4 and cN0 at diagnosis;
2. cN0 at physical examination, confirmed by axillary ultrasound (US) and magnetic resonance imaging (MRI) and fine needle aspiration cytology (FNAC) or core needle biopsy (CNB) in case of suspicious imaging;
3. Having received PST (either NACT or neoadjuvant endocrine therapy (NET) ± anti-HER2 agents) outside of clinical trials;
4. Having received a minimum of 80% of the planned cumulative dose of NACT or at least 4 months of NET;
5. Resulting SLN-positive after PST;
6. Undergoing ALND (at least Berg levels I and II) as a result of positive SLNB at the time of surgery.

Data cutoff was set at December 2021. Patients had received NACT or NET ± anti-HER2 according to main international and local guidelines.^1,14,27^ Based on this, NET instead of NACT was administered if patients were affected by hormone receptor-positive (HR+)/HER2-negative(−) BC and, despite indication to receive PST (e.g., locally advanced disease, need for shrinkage to allow conservative surgery, etc.), presented with at least one of the following: age > 80 years old, refused NACT, had contraindications to chemotherapy (CT), their tumor was Luminal A and with low/intermediate risk of recurrence (ROR) at Prosigna or Luminal B with low/intermediate ROR at Prosigna but Luminal A-like at immunohistochemistry (IHC).^28^ Any metastatic, inflammatory (cT4d) or cN+ BC patient at diagnosis or after PST was excluded from this observational study, as well as cN0 patients not complying with all inclusion criteria. Previous history of breast or axillary surgery were other exclusion criteria. This study was approved by the Ethics Committee of the HCB (reg. HCB/2021/1052). Operational procedures and pathologic assessment details are more extensively reported in the Supplementary Methods.

### Study Objectives and Statistical Analyses

The objectives of this study were (1) to describe the main clinico–pathological features of patients with EBC with baseline cN0 undergoing PST and showing postneoadjuvant SLN+, to potentially identify characteristics associated to non-SLN+ at subsequent ALND, and (2) to develop a predictive model of non-SLN+ to avoid potentially unnecessary and harmful ALND.

Continuous variables were described by mean with standard deviation (SD), whilst categorical variables by proportions. Chi-squared and Student’s *t*-tests were carried out to compare the distribution of main clinic–pathological variables in the groups of interest. Preliminary univariate and multivariate logistic regression analyses were performed to detect clinic–pathological factors associated with the presence of residual disease in non-SLN at ALND. Results were reported using odds ratios (OR) with 95% confidence intervals (CI). A logistic least absolute shrinkage and selection operator (LASSO) regression was then run with all clinic–pathological factors to identify variables with non-zero coefficients to include in a new multiparametric predictor score of non-SLN+ at ALND (ALND-predict).^29^ Cases with missing values in at least one variable were removed. The minimum *λ* penalizing factor was adopted for the purpose. The estimated coefficients of the selected variables were used to derive an unscaled score which was further scaled 0–100 to obtain the final ALND-predict score.^30,31^ A receiver operating characteristic (ROC) curve analysis was performed to evaluate the area under curve (AUC) with 95% CI of the model and the Youden Index was calculated to identify an optimal cutoff point.^30,31^ The AUC gives an indication of the discriminatory performance of the model. An AUC of 0.5 indicates no discriminative performance, whereas an AUC of 1.0 indicates perfect discrimination.^31^ A calibration of the ALND-predict continuous and dichotomic models was performed with the Hosmer–Lemershow test with *g* > *p* + 1.^32^ Bootstrap analysis with calculation of bias-corrected and accelerated (BCa) 95% CI was undertaken to validate the predictors in artificial testing cohorts after 5000 resamplings.^33,34^ The ALND-predict continuous and dichotomic models were compared using the Akaike Information Criterion (AIC).^35^ The model with the lowest AIC (at least 2 AIC unit difference for significance)^35^ was selected for further validation.

Significance was set at *p* < 0.05 and Holms–Bonferroni corrections were performed to account for multiplicity, when appropriate. All data were analyzed with R vers. 3.4.1 and SPSS vers. 24 for Mac OSX.

## Results

### Study Population Characteristics

**Fig. 1:**
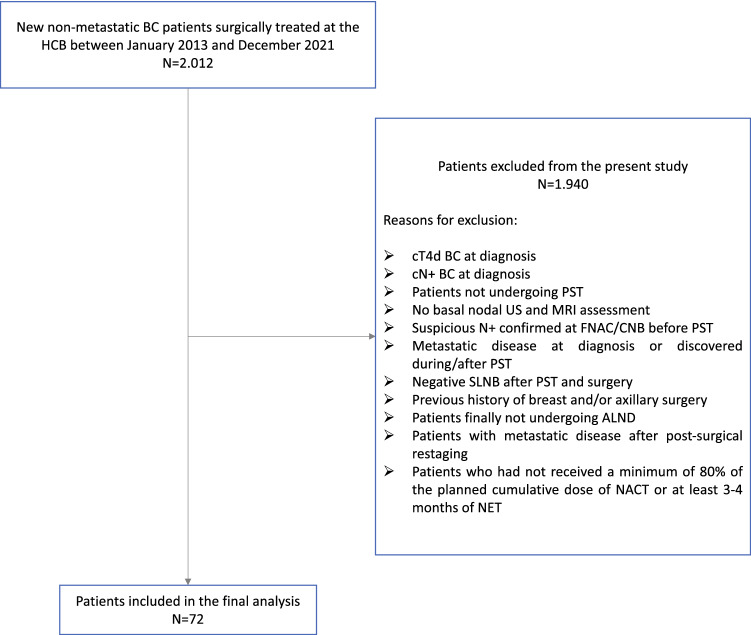
STROBE flow-chart. *BC* breast cancer, *HCB* hospital clinic of Barcelona, *c* clinical, *T4d* inflammatory BC, *US* ultrasound, *MRI* magnetic resonance imaging, *SLN* sentinel lymph node, *SLNB* sentinel lymph node biopsy, *PST* primary systemic treatment, *ALND* axillary lymph node dissection, *NACT* neoadjuvant chemotherapy, *NET* neoadjuvant endocrine therapy, + positive, *FNAC* fine needle biopsy, *CNB* core needle biopsy

Of the 2012 newly diagnosed nonmetastatic BC patients surgically treated at our hospital between January 2013 and December 2021, we included a total of 72 patients with non-inflammatory cT1–4 cN0 EBC who were treated with PST outside of clinical trials, underwent breast surgery and SLNB, showed positive SLN and received subsequent ALND (Fig. 1). All patients were Caucasian women and 61.1% were postmenopausal. Basal mean tumor dimension at magnetic resonance imaging (MRI) was 32.5 mm (SD ± 16.5 mm), most BC were of ductal histology (86.1%), grade (G)II/III (83.3%), and Ki67–high (81.8% > 14%). Overall 76.4% were HR+/HER2−, 12.5% HER2-positive (+), and 11.1% triple negative (TNBC). Population characteristics are fully described in Table 1.

**Table 1:** Overall population demographics and distribution according to ALND results

Demographics	Overall population		Non-SLN negative		Non-SLN positive		χ^2^ *p* value	Uni log reg *P*
	*N*	%	*N*	%	*N*	%		
	72	100.0	56	77.8	16	22.2		
*Age at diagnosis (years)*								
Mean	55.5	–	56.2	–	53.1	–	0.366	0.472
SD	± 13.5	–	± 14.01	–	± 11.5	–		
*BMI (kg/m*^2^*)*								
Mean	25.4	–	24.9	–	27.4	–	0.106	0.572
SD	± 4.8	–	± 4.4	–	± 5.6	–		
*Menopause at diagnosis*								
Yes	44	61.1	35	62.5	9	56.3	0.651	0.652
No	28	38.9	21	37.5	7	43.8		
*Primary tumor size (mm) according to MRI*								
Mean	32.5	–	31.9	–	34.6	–	0.633	0.576
SD	± 16.5	–	± 15.5	–	± 19.8	–		
*Primary tumor size (clinical TNM category)*								
cT1 (0.1–20.0 mm)	17	23.6	15	26.8	2	12.5	0.494	0.576°
cT2 (20.1–50.0 mm)	43	59.7	32	57.1	11	68.8		
cT3 (>50.0 mm)/cT4 (non-inflammatory)	12	16.7	9	16.1	3	18.8		
*Unconfirmed suspicious cN+ at diagnosis*^a^								
Yes	16	22.2	10	17.9	6	37.5	0.096	0.103
No	56	77.8	46	82.1	10	62.5		
*Histology type*								
Ductal	62	86.1	50	89.3	12	75.0	0.145	0.157
Lobular/other	10	13.9	6	10.7	4	25.0		
*Tumor grade*								
I	12	16.7	9	16.1	3	18.8	0.801	0.975*
II	47	65.3	36	64.3	11	68.8		
III	13	18.1	11	19.6	2	12.5		
*Ki67%*								
Mean	27.8	–	30.4	–	20.9	–	0.058	0.086
SD	±17.1	–	±17.5	–	±14.1	–		
≤14%	12	18.2	6	11.8	6	40.0	**0.013**	**0.018**
>14%	54	81.8	45	88.2	9	60.0		
Overall	66	91.7	51	91.1	15	93.8		
*ER%*								
Mean	72.6	–	69.7	–	82.4	–	0.096	0.225
SD	±36.0	–	±38.6	–	±23.6	–		
*PR%*								
Mean	49.2	–	43.2	–	70.3	–	**0.015**	**0.024**
SD	±40.5	–	±40.1	–	±35.6	–		
*IHC tumor classification*								
HR+/HER2–	55	76.4	41	73.2	14	87.5	0.493	0.248
HER2+	9	12.5	8	14.3	1	6.3		
TN	8	11.1	7	12.5	1	6.3		
*Tumor focality*								
Unifocal	41	56.9	33	58.9	8	50.0	0.525	0.526
Multifocal/multicentric	31	43.1	23	41.1	8	50.0		
*Primary systemic treatment*								
Endocrine therapy (AI or tamoxifen)	17	23.6	10	17.9	7	43.8	**0.031**^**b**^	**0.038**^**b**^
Anthracyclin + Taxanes +/– anti-HER2	44	61.1	37	66.1	7	43.8		
Taxanes +/– anti-HER2	11	15.3	9	16.1	2	12.5		
*Breast MRI response after PST*								
Progression	1	1.4	1	1.8	0	0.0	0.305	0.105
Partial response ≤50% + Stable Disease	32	45.1	22	40.0	10	62.5		
Partial response >50%	24	33.8	19	34.5	5	31.3		
Complete response	14	19.7	13	23.6	1	6.3		
Overall	71	98.6	55	98.2	16	100.0		
*Type of surgery*								
Conservative	29	40.3	24	42.9	5	31.3	0.404	NE
Mastectomy	43	59.7	32	57.1	11	68.8		
*SLN evaluation*								
Conventional	17	23.6	11	19.6	6	37.5	0.138	0.145
OSNA	55	76.4	45	80.4	10	62.5		
*Number of positive SLN*								
Mean	1.5	–	1.4	–	1.9	–	**0.039**	**0.019**
SD	±0.7	–	±0.6	–	±0.8	–		
*Type of positive SLN*								
ITC/micrometastasis	37	51.4	34	60.7	3	18.8	**0.003**	**0.006**
Macrometastasis	35	48.6	22	39.3	13	81.3		

Signficant *p* values are reported in bold

^*^Grade I versus grade II/III

^a^US/MRI/clinical suspicious nodes at diagnosis, unconfirmed at biopsy/cytology, °MRI cT ≤3 cm versus MRI cT >3 cm; *cT* clinical primary tumor dimension, *cN* clinical axillary lymph-nodes status

^b^Neoadjuvant chemotherapy versus endocrine therapy

*ALND* Axillary lymph node dissection, *SLN* Sentinel lymph node, *uni log reg* univariate logistic regression, *SD* Standard deviation, *US* Ultrasound, *MRI* Magnetic resonance imaging, *ER* Estrogen receptor, *PR* Progesterone receptor, *HER2−* HER2 negative, *HER2+* HER2 positive, *HR+* Hormone receptor positive, *TN* Triple negative, *AI* Aromatase inhibitor, PST Primary systemic therapy, *IHC* Immunohistochemical, *OSNA* One-step nucleic acid amplification, *ITC* Isolated tumor cells, *NE* Not evaluated

**Fig. 2:**
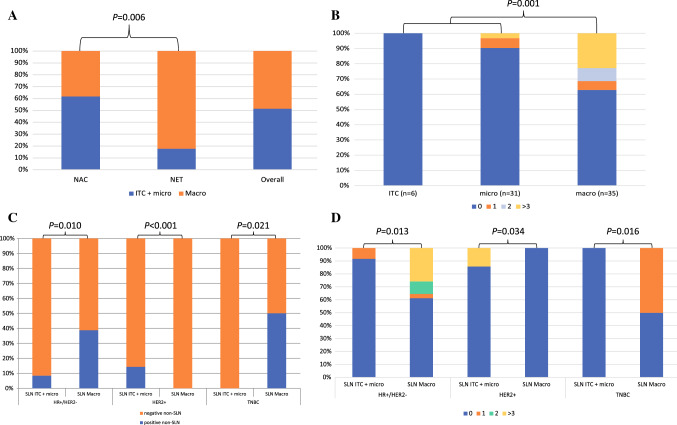
Patterns of SLN and non-SLN involvement. **A** Pattern of SLN involvement according to primary systemic therapy and in the overall population. **B** Number of non-SLN affected after ALND, according to SLN involvement type. **C** Type of non-SLN affected after ALND, according to SLN involvement within each IHC subtype. **D** Number of non-SLN affected after ALND, according to SLN involvement within each IHC subtype. *IHC* immunohistochemistry, *SLN* sentinel lymph node, *ALND* axillary lymph node dissection, *NAC* neoadjuvant chemotherapy, *NET* neoadjuvant endocrine therapy, *ITC* isolated tumor cells, *Micro* micrometastases, *Macro* macrometastases, *HR* hormone receptor, + positive, − negative, *TNBC* triple negative breast cancer. *p* values refer to Chi-squared tests

The majority of patients (61.1%) received a PST with anthracycline + taxane-based NACT, while 15.3% received a taxane-based NACT and 23.6% received NET with aromatase inhibitors (AI) or tamoxifen (median duration: 6 months; min–max range: 4–9 months). All patients with HER2+ BC also received anti-HER2 agents. After PST, conservative breast surgery was undertaken in 40.3% patients. All patients within this study had a positive SLNB and the mean number of positive nodes was 1.5 (SD ± 0.7), with macrometastases observed in 48.6% of cases (Table 1). Of note, patients receiving NET were more frequently found with macrometastases (82.4% vs. 38.2%) and with no ITC (0% vs. 10.9%), compared with patients receiving NACT (*p* = 0.006) (Fig. 2). Regional nodal RT after surgery was then administered in 68.1% cases.

In this population, in-breast pCR, meaning absence of infiltrating BC in the surgical specimen, was achieved in 13 (18.1%) cases. After a median follow-up of 46.2 months (95% CI: 41.0–56.6 months), only three events in three different patients were detected in the form of bone metastasis (two in TNBC, one in HR+/HER2− BC) and only in one of these patients additional positive nodes after ALND had been detected.

A separate descriptive evaluation of the HR+/HER2− subpopulation undergoing NET is reported in the Supplementary Materials.

### Association between Clinico–Pathological Factors and Non-SLN+ at ALND

Overall, 56 (77.8%) patients presented without non-SLN involvement (non-SLN−) after ALND, while 16 (22.2%) showed at least one infiltrated non-SLN. Patients with non-SLN− and non-SLN+ did not differ in many clinic–pathological factors (Table 1). However, at univariate logistic regression analysis, PR levels (continuous, OR: 1.02, 95% CI: 1.00–1.04, *p* = 0.024), Ki67 (≤ 14% vs. >14%, OR: 5.33, 95% CI: 1.40–20.31, *p* = 0.014), PST type (NACT vs. NET, OR: 0.28, 95% CI: 0.08–0.93, *p* = 0.038), the number of positive SLN (continuous, OR: 2.55, 95% CI: 1.17–5.57, *p* = 0.019; 1 versus 2–3, OR: 0.28, 95% CI: 0.09–0.90, *p* = 0.033), and the type of SLN involvement (ITC/micrometastases versus macrometastases, OR: 0.15, 95% CI: 0.04–0.59, *p* = 0.006) were significantly associated with non-SLN+ (Table 1). These associations were reflected by the significant differences observed between non-SLN− and non-SLN+ population characteristics (Table 1 and Fig. 2).

On multivariate logistic regression, only increasing PR levels (*p* = 0.031) and macrometastatic SLN+ (*p* = 0.040) were significantly and independently associated with non-SLN+ after ALND (Table 2). Macrometastases were usually associated to non-SLN+ and a higher number of non-SLN+ in HR+/HER2− (*p* = 0.010 and *p* = 0.013, respectively) and TNBC *(p* = 0.021 and *p* = 0.016, respectively) (Fig. 2), while the opposite association was observed within the HER2+ subgroup (*p* < 0.001 and *p* = 0.034, respectively) (Fig. 2).

**Table 2:** Multivariate logistic regression models for the primary and secondary endpoints

Variables	OR	Inferior 95% CI	Superior 95% CI	*p* value
Menopausal status (Post versus Pre)	0.97	0.17	5.63	0.972
IHC subtypes (HR+/HER2− versus HER2+/TNBC)	0.37	0.02	8.70	0.535
PgR% (continuous)	**1.03**	**1.00**	**1.06**	**0.031**
Ki67% (≤14% versus >14%)	1.41	0.12	16.75	0.785
cT at MRI (continuous, in mm)	1.02	0.97	1.06	0.445
Radiologic response (complete/partial versus stable/progression)	0.84	0.16	4.31	0.835
PST (NACT versus NET)	1.13	0.11	11.25	0.918
SLN+ number (continuous)	1.60	0.54	4.72	0.398
SLN+ type (ITC/micrometastatic versus macrometastatic)	**0.10**	**0.01**	**0.90**	**0.040**
Suspicious cN+ (clinico–radiological suspect* versus no suspect)	0.13	0.02	1.05	0.055

Variables	OR	Inferior 95% CI	Superior 95% CI	*p* value
ALND-predict (continuous)	**1.06**	**1.02**	**1.10**	**0.002**
cT at MRI (continuous, in mm)	1.01	0.97	1.05	0.634
PST (NACT versus NET)	1.27	0.22	7.50	0.791
Radiologic response (complete/partial versus stable/progression)	0.80	0.19	3.20	0.760
SLN assessment technique (conventional versus OSNA)	1.61	0.37	6.97	0.521
Age (≤50 versus >50 years)	1.51	0.35	6.54	0.585

Variables	OR	Inferior 95% CI	Superior 95% CI	*p* value
ALND-predict (≥ cutoff versus < cutoff)	**23.77**	**4.10**	**137.80**	**< 0.001**
IHC subtypes (HR+/HER2− versus HER2+/TNBC)	0.84	0.10	6.79	0.870
cT at MRI (continuous, in mm)	1.01	0.96	1.06	0.679
PST (NACT versus NET)	1.11	0.18	7.06	0.910
Radiologic response (complete/partial versus stable/progression)	0.68	0.15	3.02	0.612
SLN assessment technique (conventional versus OSNA)	1.62	0.33	8.08	0.556
Age (≤50 versus >50 years)	2.77	0.57	13.58	0.209

Significant *p* values are reported in bold

^*^Unconfirmed at biopsy/cytology

*SLN* Sentinel lymph nodes, *post* Postmenopausal, *pre* Premenopausal, *PR* Progesterone receptor, *IHC* Immunohistochemistry, *HR* Hormone receptor, *+* Positive, *−* Negative, *TNBC* Triple negative breast cancer, *ITC* Isolated tumor cells, *MRI* Magnetic resonance imaging, *cT* Clinical primary tumor dimension, *cN* Clinical axillary lymph node status, *OR* Odds ratio, *CI* Confidence interval, *PST* Primary systemic therapy, *NACT* Neoadjuvant chemotherapy, *NET* Neoadjuvant endocrine therapy, *ALND* Axillary lymph node dissection

### Building the ALND-Predict Multiparametric Score

**Fig. 3:**
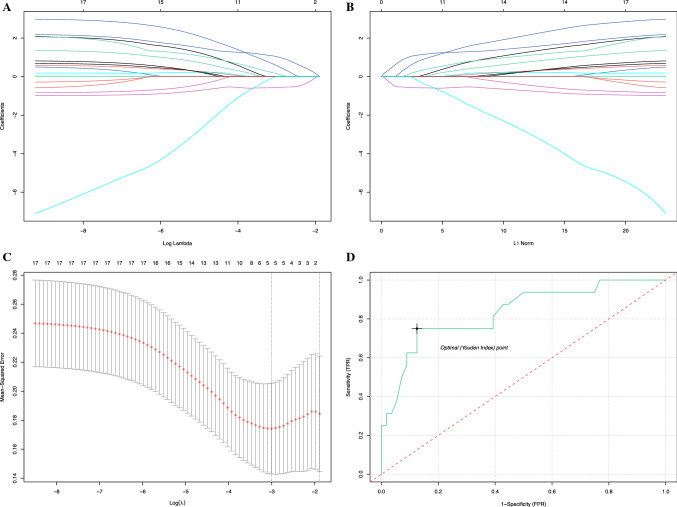
Demographic and clinical feature selection using the LASSO binary logistic regression model and ROC curve of the final ALND-predict model. **A** and **B** Plots of the beta coefficient paths, representing the optimal parameter (λ) selection in the LASSO model. A cross-validation via minimum criteria was used. Each colored line represents the value taken by a different coefficient in the model. The partial likelihood deviance (binomial deviance) curve was plotted versus log(λ) in A, and the L1 Norm in B. λ is the weight given to the regularization term (the L1 norm) of the LASSO function. When λ is very small, the LASSO solution should be very close to the ordinary least square (OLS) solution, and all the coefficients are included in the model. In A, this is represented by smaller log(λ) values on the *x* axis being associated to higher number of variables entering the model. The *x* axis in B is the maximum permissible value the L1 Norm can take. Smaller L1 Norm values (left section of B) correspond to higher regularization, implying less variables with non-zero coefficients entering the model. **C** LASSO coefficient profiles of the variables. A coefficient profile plot was produced against the log(λ) sequence. Vertical line was drawn at the value selected using fivefold cross-validation, where optimal λ resulted in five features with nonzero coefficients. Each red dot is a λ value, with respective standard error (SE) depicted by the gray whiskers. The numbers on top are the number of nonzero regression coefficients in the model corresponding to each λ. From left to right along the *x* axis, with increasing fewer variables are included in the model, since the penalty for inclusion of features is weighted more heavily. The dashed lines are the log values corresponding to the λmin (left dashed line) and λ_1SE_ (right dashed line). **D** ROC curve of the ALND-predict model, with optimal cutoff point by the Youden Index. *LASSO* least absolute shrinkage and selection operator, *TPR* true positive rate, *FPR* false positive rate, *ROC* receiver operating characteristics

We carried out a LASSO regression and extracted the non-zero coefficients of the variables ultimately identified (i.e., PR levels, dichotomized Ki67, number of SLN+, and type of SLN+ involvement) (Fig. 3), with the exception of “suspicious unconfirmed nodal involvement,” which was excluded to mitigate nonreproducibility issues [the suspicion mostly relied on ultrasonographic (US) evaluation]. Then, we calculated an unscaled predictive score, according to the following formula: (0.00202 × PR%) + (− 0.127 × Ki67 as dichotomic) + (0.0517 × number of positive SLN) + (0.165 × SLN+ type as dichotomic). We further rescaled the score (original values range: − 0.075, +0.4817) to reflect a range from 0 to 100 points and named it the ALND-predict score. The AUC for predicting non-SLN+ was 0.83 (95% CI: 0.71–0.96). We also built an alternative model with all LASSO-identified variables. The AUC difference with the ALND-predict was 0.009 (95% CI: − 0.031, 0.049) and nonsignificant for De Long *p* = 0.666. Thus, we retained the four-variable ALND-predict. An optimal cutoff based on the Youden Index was found to be 63, with a sensitivity of 0.75 (95% CI: 0.48–0.93) and a specificity of 0.88 (0.76–0.95) (Fig. 3). The positive predictive value (PPV) was 0.63 (0.44–0.88) and the negative predictive value (NPV) was 0.92 (95% CI: 0.79 0.97). Both ALND-predict as continuous score and dichotomic predictor passed the Hosmer–Lemershow test for the goodness of fit (*p* = 0.876 and *p* = 1.00, respectively).

ALND-predict was significantly associated with non-SLN+ on univariate analysis as continuous (OR: 1.06, 95% CI: 1.03–1.09, *p* < 0.001) and dichotomic variable (above/equal versus below the optimal cutoff (OR: 21.0, 95% CI: 5.28–83.57, *p* < 0.001). The association was confirmed at multivariate analysis for both the continuous (OR: 1.06, 95% CI: 1.02–1.10, *p* = 0.002) and dichotomic predictor (OR: 23.77, 95% CI: 4.10–137.80, *p* < 0.001), independently from age, IHC subtype, primary tumor dimension before PST, radiological response to PST, type of PST, and SLN assessment technique. Conversely, the other variables were not significant (Table 2). The variables included in the predictor were excluded to avoid multicollinearity. However, the AUC of the model outperformed the AUC of all its single variables **(**Supplementary Fig. 1).

On the 5000 bootstrap-adjusted retesting of the multivariate models, estimated BCa 95% CI for the continuous (1.01–1.11) and dichotomic (1.61–319.58) predictor included the adjusted OR in both cases.

The dichotomic ALND-predict model provided superior predictive information compared with the continuous score [AIC 57.37 versus 60.58, respectively (difference > 2)].

In addition, we reapplied the ALND-predict only to the subset of NACT-treated patients to evaluate its performance in this context, as NACT is more frequently carried out than NET. The predictor identified three false positives (37.5% of all patients identified as non-SLN+) and four false negatives (8.5% of all patients identified as non-SLN−). For exploratory purposes, we also rebuilt the predictor by only using the NACT-treated subset of patients. However, we ultimately decided to retain the original ALND-predict (Supplementary Results). The two models, constructed on different datasets and including different parameters, despite being both associated to non-SLN+ when above the respective predefined cutoffs, did not perform the same. In fact, the newer predictor showed that this might lead to more ALND in a context where this could represent an overtreatment. Yet both showed a false negative rate below 10% (Supplementary Results).

## Discussion

In the neoadjuvant setting of EBC, defining the optimal axillary management in SLN+ patients with cN0 EBC at baseline is a relevant unmet need. Noteworthy, studies involving baseline cN0 with SLN+ after postneoadjuvant SLNB have usually included baseline cN+ and/or cN0 postneoadjuvant SLN-negative cases, jeopardizing the evidence accumulated so far.^36,37^ Furthermore, additional axillary lymph node involvement after NACT has been reported in up to 60% cases.^38,39^ Consequently, ALND is usually recommended by most guidelines in this scenario.^1,14,40,41^ In this perspective, we investigated non-SLN+ rates after ALND in a pure cohort of non-inflammatory cT1–4 cN0 EBC patients with SLN+ following PST (either NACT or NET). The mean number of positive nodes was 1.5, with approximately half ITC/micrometastases and half macrometastases. NACT was generally more effective than NET in reducing the rate of macrometastatic SLN. Only increasing PR levels and macrometastatic SLN+ were significantly and independently associated with non-SLN+ after ALND. Notably, we also observed that SLN with macrometastatic dissemination and a higher number of lymph nodes involved, were more frequently associated to non-SLN+ in HR+/HER2− and TNBC. Unexpectedly, the opposite association was observed within the HER2+ subgroup. Whether this was related to differential PST effects, different tumor biology, or chance is unclear, since too few cases prevented us from drawing definitive conclusions.

However, metastatic non-SLN after ALND were found in only 22.2% of patients and only three distant recurrences were detected. Moreover, additional non-SLN+ at ALND had only been detected in one case, suggesting that ALND might be spared in a relevant proportion of initially cN0 EBC treated with PST. Notably, tumor relapses in our cohort seemed to be more related to BC IHC-defined molecular subtypes rather than axillary node status (two out of three distant recurrences were in TNBC patients). Nevertheless, the limited cohort prevents us from drawing clear conclusions regarding this aspect.

Our ALND-predict multiparametric score based on PR, Ki67, and SLN+ status, was significantly and independently associated, either as continuous (value range 0–100) or dichotomic variable (cutoff of 63), with non-SLN status. More specifically, in patients with SLN+ after PST (NACT or NET) and tumor surgery, the odds of presenting non-SLN+ at ALND were 6% higher for each unitary increase in the ALND-predict score. Furthermore, a value ≥ 63 was able to identify the majority of non-SLN+ patients at ALND and, importantly, almost all negative cases. The sensitivity was 75%, the specificity was 87.5%, the PPV was 63.2%, and the NPV was 92.5%. This means that in case of SLN+ EBC patients after PST, the predictor would be able to effectively identify almost all patients with truly negative non-SLN, with only a 7.5% chance of missing patients with non-SLN+ if ALND were performed. This is remarkable, also considering the low proportion of non-SLN+ observed after subsequent ALND, the high risk of lymphedema and other morbidities, as well as the uncertainties regarding the true impact on local/distant relapse risk reduction, which seems to be relatively low, at least in our cohort. Moreover a 7.5% chance of undetected true negative would be lower than the false negative rate currently considered acceptable for SLN (< 10%).^42,43^ This result was confirmed to be independent from patients’ age, tumor IHC subtype (HR+/HER2−, HER2+, TNBC), clinical stage at diagnosis, PST (NACT or NET, with or without anti-HER2 agents), radiologic response to PST, and SLN study methods [conventional or One-Step Nucleic Acid (OSNA)]. Furthermore, the predictor model outperformed all the single variables that it integrated. If results for ALND-predict are further confirmed, its subsequent implementation in the clinic might pave the way toward an ALND-sparing approach in baseline non-inflammatory cT1–4 cN0 EBC treated with PST, and resulting SLN+ after tumor surgery and SLNB.

Our study presents some limitations. First, it is debated whether non-SLN+ is truly prognostically unfavorable in neoadjuvant-pretreated EBC, and we could not properly test this hypothesis in our cohort. Second, SLN involvement was assessed in most cases (>75%) with the One-Step Nucleic Acid (OSNA) technique.^44^ Hence, the presence of extracapsular nodal involvement was not uniformly reported and could not be included as a variable in our regression models. Third, the study is retrospective in its nature. Nevertheless, all patients had been consecutively enrolled in a surgical prospective observational database, hence all patients complying with inclusion criteria were included in our study. In addition, despite a reduced number of patients, the population of interest is extremely selected for its characteristics, with no available and detailed data published so far. Only 24–32% of patients with cN0 EBC are SLN+ after PST, and results from other studies are mixed with BC patients with other features, such as cN+ at diagnosis before receiving PST or negative SLN after PST.^36,37^ However, positive or negative SLN after PST may have different prognostic implications, also depending on whether patients were initially classified as cN0 or cN+. At present, the most solid evidence on ALND-sparing approaches are relegated to EBC with cT1–2 cN0 BC undergoing primary surgery and subsequent adjuvant treatments.^21,22,25,26,45,46^ Importantly, axillary management in cN0 patients at diagnosis with low SLN tumor burden (ITC/micrometastasis) after PST was subject to controversy in the latest St. Gallen International Consensus. Many panelists felt axillary RT could be an alternative to axillary dissection in such situations. Other panelists urged caution, noting persistent risks of residual axillary nodal involvement, and recommended awaiting the results of ongoing phase III trials.^1,47^

A major limitation of ALND-predict is that it was built on a cohort that included HR+/HER2− BC patients treated with NET. This PST approach is not as widespread as NACT and the role of ALND in case of SLN+ after NET is even less clear in this scenario. Hence, we exploratorily rebuilt the predictor model by removing NET-receiving patients at the time of performing the LASSO regression for the variables selection, so to obtain an alternative predictor. Nevertheless, we preferred to ultimately retain the original ALND-predict because (1) it was associated with potentially less ALND in a pure NACT-treated population maintaining the false negative rate below the currently accepted cut-off of 10%;^42,43^ (2) it took into account different neoadjuvant therapeutic approaches (NACT versus NET) when constructing the model, making it more generalizable to institutions performing NET; and (3) it was built on a higher number of patients. To note, we also reapplied the original ALND-predict only to patients undergoing NACT, confirming a false negative rate of < 10%.

Finally, we had no validation cohort for the ALND-predict. However, we derived the multiparametric model from centrally reviewed parameters (all assessed at the HCB), with an optimal nodal staging to define cN0 at diagnosis (clinical, US-, and MRI-based), with a robust statistical methodology, and provided an internal validation with a 5000 bootstrap-adjusted retesting of both the continuous and dichotomic predictors, which is a widely accepted methodology for validating logistic regression-based predictive models in the absence of external cohorts.^33,49,50^ While this does not necessarily imply the possibility of a direct clinical implementation of the predictor, it highlights a remarkably stable accuracy in detecting patients to whom ALND should not be offered. Furthermore, the model is easily appliable to almost every clinical practice scenario, being cheap and based on parameters routinely available in the setting of interest. Therefore, ALND-predict merits further prospective validation.

## Supplementary Information

Below is the link to the electronic supplementary material.

Supplementary file1 (DOCX 288 KB)
