# Supplementary material for: Nonsentinel Axillary Lymph Node Status in Clinically Node-Negative Early Breast Cancer After Primary Systemic Therapy and Positive Sentinel Lymph Node: A Predictive Model Proposal
Source: Ann Surg Oncol. 2023 Feb 21;30(8):4657–68. doi: 10.1245/s10434-023-13231-x (PMC10319670; doi:10.1245/s10434-023-13231-x)
Supplement: Supplementary file 1 — Supplementary file1 (DOCX 288 KB) [file 10434_2023_13231_MOESM1_ESM.docx]

SUMMARY

[Supplementary methods 2](#_Toc122453049)

[Study procedures 2](#_Toc122453050)

[Pathologic Examination 3](#_Toc122453051)

[Supplementary References 3](#_Toc122453052)

[Supplementary Results 5](#_Toc122453053)

[Focus on the NET subset 5](#_Toc122453054)

[Alternative score in the NACT population 6](#_Toc122453055)

[Supplementary tables 8](#_Toc122453056)

[Supplementary table 1. Main characteristics and outcomes of the NET subpopulation 8](#_Toc122453057)

[Supplementary figures 10](#_Toc122453058)

[Supplementary figure 1. ROC curves of the variables integrating the ALND-Predict multiparametric score showing their capability of detecting patients with non-SLN+ at ALND 10](#_Toc122453059)

# Supplementary methods

## **Study procedures**

As *per* standard clinical practice at the Hospital Clinic of Barcelona (HCB), patients diagnosed with invasive breast cancer (BC) were evaluated by a breast surgeon who clinically explored the axilla. All patients underwent axillary ultrasound (US) assessment and locoregional breast and axillary study by magnetic resonance imaging (MRI). The few patients who had not undergone clinical plus radiological axillary evaluation before PST were excluded from this analysis.

Criteria for cN0 previous to PST were negative physical exploration accompanied by negative findings on axillary US and MRI, described as no visible lymph nodes (LN) in axilla or LN with cortical thickness <3 mm at US. When suspicious lymph nodes were identified at imaging study, fine needle aspiration cytology (FNAC) or core needle biopsy (CNB) were performed to exclude N+ disease, as *per* standard practice.

The overall therapeutic strategy was discussed at the Multidisciplinary Tumor Board and PST selection between neoadjuvant chemotherapy (NACT) or endocrine therapy (NET), as well as the type of regimen administered were decided by the HCB medical oncologists based on tumors’ clinico-pathological features and patient’s characteristics, preferences and comorbidities, according to HCB practice and main international guidelines^1–3^.

Patients included in final study analysis received a minimum of 80% of the planned cumulative dose of NACT or at least 4 months of NET. During the duration of PST, patients were usually evaluated at 2-3 week intervals by the medical oncology team and treatment schedules modified based on intercurrent toxicities, if necessary.

After completion of PST, a second MRI was usually performed between 4-6 weeks before surgery to assess radiological tumor response to systemic treatment. RECIST 1.1 criteria were used for assessing tumor response after PST^4^. Post-neoadjuvant SLNB was performed in all patients by a peritumoral injection of 11 MBq of technetium 99m-nanocolloid the day before surgery and subsequent lymphoscintigraphy to check SLN drainage at the axillary level. The day of surgery, SLN was pursued and retrieved using a hand-held gamma probe. Anatomopathological assessment of the SLN was performed in all patients of the study, either by the conventional method or by One-Step Nucleic Acid Amplification (OSNA) technique. The conventional method consists in an intraoperative study of touch imprint cytology and frozen section, followed by differed study with hematoxylin-eosin and cytokeratin immunohistochemistry (IHC) staining, reserved for cases when the study of cytokeratin 19 was negative or unknown. Several studies have supported the concordance of the OSNA technique with conventional SLN detection, both in the adjuvant and neoadjuvant context^5–8^. The assay was elsewhere described in detail^9^ and provides correlation among cytokeratin 19 (CK19) mRNA copies/μL and SLN metastasis size, as follows: isolated tumor cells (ITC) for <2.5 x 10^2^ copies/μL; micrometastasis for 2.5 x 10^2^ - 5 x 10^3^ copies/μL; macrometastasis, for >5 x 10^3^ copies/μL^9^.

All patients in this study had positive SLNB and underwent axillary lymph-node dissection (ALND). Qualitative and quantitative information on SLN and non-SLN removed were collected.

## **Pathologic Examination**

IHC evaluation of tumor grade (G), Ki67, estrogen receptor (ER) and progesterone receptor (PR) status was carried out as also described elsewhere ^10^ on diagnostic biopsy samples. For the purpose of this analysis, hormone receptor (HR) status was defined as positive (+) if ER and/or PR were ≥1%, according to the latest ASCO/CAP guidelines^11^. Human epidermal growth factor receptor 2 (HER2) status was assessed according to the ASCO/CAP guidelines available at the time of patient’s diagnosis^12–14^. Tumors were classified based on HR and HER2 status as HR+/HER2-negative (-), HER2+ (independently from HR status), or triple negative breast cancer (TNBC), if HR-/HER2.

## **Supplementary References**

1 Burstein HJ, Curigliano G, Thürlimann B, *et al.* Customizing local and systemic therapies for women with early breast cancer: the St. Gallen International Consensus Guidelines for treatment of early breast cancer 2021. *Ann Oncol* 2021; **32**: 1216–35.

2 Cardoso F, Kyriakides S, Ohno S, *et al.* Early breast cancer: ESMO Clinical Practice Guidelines for diagnosis, treatment and follow-up. *Ann Oncol* 2019; **30**: 1674.

3 Burstein HJ, Somerfield MR, Barton DL, *et al.* Endocrine Treatment and Targeted Therapy for Hormone Receptor-Positive, Human Epidermal Growth Factor Receptor 2-Negative Metastatic Breast Cancer: ASCO Guideline Update. *J Clin Oncol* 2021; : JCO2101392.

4 Eisenhauer EA, Therasse P, Bogaerts J, *et al.* New response evaluation criteria in solid tumours: revised RECIST guideline (version 1.1). *Eur J Cancer* 2009; **45**: 228–47.

5 Bernet L, Cano R, Martinez M, *et al.* Diagnosis of the sentinel lymph node in breast cancer: a reproducible molecular method: a multicentric Spanish study. *Histopathology* 2011; **58**: 863–9.

6 Osako T, Tsuda H, Horii R, *et al.* Molecular detection of lymph node metastasis in breast cancer patients treated with preoperative systemic chemotherapy: a prospective multicentre trial using the one-step nucleic acid amplification assay. *Br J Cancer* 2013; **109**: 1693–8.

7 Navarro-Cecilia J, Dueñas-Rodríguez B, Luque-López C, *et al.* Intraoperative sentinel node biopsy by one-step nucleic acid amplification (OSNA) avoids axillary lymphadenectomy in women with breast cancer treated with neoadjuvant chemotherapy. *Eur J Surg Oncol* 2013; **39**: 873–9.

8 Parada D, Peña KB, Riu FF, Aguilar AE, Cohan S. Intraoperative molecular analysis of sentinel lymph nodes following neoadjuvant chemotherapy in patients with clinical node negative breast cancer: An institutional study. *Mol Clin Oncol* 2016; **5**: 507–10.

9 Tsujimoto M, Nakabayashi K, Yoshidome K, *et al.* One-step nucleic acid amplification for intraoperative detection of lymph node metastasis in breast cancer patients. *Clin Cancer Res* 2007; **13**: 4807–16.

10 De Angelis C, Di Maio M, Crispo A, *et al.* Luminal-like HER2-negative stage IA breast cancer: a multicenter retrospective study on long-term outcome with propensity score analysis. *Oncotarget* 2017; **8**: 112816–24.

11 Allison KH, Hammond MEH, Dowsett M, *et al.* Estrogen and Progesterone Receptor Testing in Breast Cancer: ASCO/CAP Guideline Update. *JCO* 2020; **38**: 1346–66.

12 Wolff AC, Hammond MEH, Schwartz JN, *et al.* American Society of Clinical Oncology/College of American Pathologists guideline recommendations for human epidermal growth factor receptor 2 testing in breast cancer. *J Clin Oncol* 2007; **25**: 118–45.

13 Wolff AC, Hammond MEH, Hicks DG, *et al.* Recommendations for human epidermal growth factor receptor 2 testing in breast cancer: American Society of Clinical Oncology/College of American Pathologists clinical practice guideline update. *J Clin Oncol* 2013; **31**: 3997–4013.

14 Wolff AC, Hammond MEH, Allison KH, *et al.* Human Epidermal Growth Factor Receptor 2 Testing in Breast Cancer: American Society of Clinical Oncology/College of American Pathologists Clinical Practice Guideline Focused Update. *J Clin Oncol* 2018; **36**: 2105–22.

# Supplementary Results

## **Focus on the NET subset**

We explored the characteristics of the subset of hormone receptor-positive (HR+)/HER2-negative patients treated with neoadjuvant endocrine therapy (NET). Seventeen patients overall were treated with either tamoxifen (23.5% premenopausal) or an AI (76.5% postmenopausal). These patients received 4 to 9 months of NET and were mostly (76.4%) cT1-2 at magnetic resonance imaging (MRI) at diagnosis. Five (29.4%) were of lobular histology, while the others were ductal (70.6%). No grade (G)3 tumors were observed, but most tumors were G2 (81.3%), with low Ki67 (mean of 17%, 52.9% below the cut-off of 14%) and high mean levels of estrogen receptor (ER) and progesterone receptor (PR) (mean of 90% and 70%, respectively). Responses were overall poor, in line with what have been reported in the literature. The majority experienced a stable disease at MRI (64.7%), with one progression observed during treatment and mastectomy ultimately received by 52.9% of patients. Yet, one complete response both at imaging and in terms of in-breast pathologic complete response (pCR) were observed. A mean of 2 positive lymph-nodes were found at post-neoadjuvant sentinel lyph-node biopsy (SLNB) and 82.4% presented with macrometastases. Overall, 41.2% patients showed a non-sentinel axillary lymph-node positivity (non-SLN+) at axillary dissection (ALND) after NET and surgery. Finally, no relapses nor deaths were observed. Main patients and tumors characteristics are detailed in **Supplementary table 1**. There were no significant clinicopathological differences between non-SLN+ and negative patients at ALND, with the exception of non-SLN+ showing a higher mean of positive sentinel lymph-nodes (SLN+) (2 vs. 1, p=0.021) and received more mastectomy (85.7% vs. 30.0%, p=0.024), likely due to lower responses.

Compared to the overall population included in our study, where 33.8% achieved a radiologic partial response and 19.7% a radiologic complete response at MRI, NET-treated patients showed poorer responses to the administered primary systemic treatment (PST) and the rate of non-SLN+ at ALND was also higher (41.2% vs. 22.2%). Moreover, numerically, these patients were older than what observed in the overall casuistry (a mean of 63 vs. 55.5 years) and presented tumors with lower Ki67 (mean of 28% vs. 17%) but similar rate of cT1-2 cases (approximately 80% in both cases). More lobular tumors (29.4% vs. 13.9%) pertained to the NET cohort, as well, and macrometastatic nodal involvement was more frequent (82.4% vs. 48.6%) compared to the overall population.

## **Alternative score in the NACT population**

NET is not universally adopted as neoadjuvant therapeutic strategy in endocrine sensitive BC and no standardized approach exist regarding axillary management after NET in the presence of residual disease. As such, we ultimately decided to rebuild the predictor model by removing NET-receiving patients at the time of performing the LASSO regression for the variables selection. Consequently, we removed the variable referred to the therapeutic approach (NACT vs. NET) from the LASSO model. As expected, we obtained a different outcome.

**Legend.** Plot of the beta coefficient paths, representing the optimal parameter (λ) selection in the LASSO model. A cross-validation via minimum criteria was used. Each colored line represents the value taken by a different coefficient in the model. The partial likelihood deviance (binomial deviance) curve was plotted versus log(λ). λ is the weight given to the regularization term (the L1 norm) of the LASSO function. When λ is very small, the LASSO solution should be very close to the Ordinary Least Square (OLS) solution, and all the coefficients are included in the model. In this picture, this is represented by smaller log(λ) values on the x-axis being associated to higher number of variables entering the model.

Namely, the parameters to include in the alternative predictor were: menopausal status (pre/post), PgR levels (continuous), suspect of positive axillary nodes at diagnosis (yes/no), SLN positivity (yes/no) and in-breast pCR (yes/no).

As a consequence the posterior analytical validation of this alternative ALND-Predict differed completely. We obtained an unscaled continuous predictor, which we scaled 0-100 as previously done with the main ALND-Predict. Subsequently, ROC curve analysis was performed to evaluate the area under curve (AUC) with 95% confidence interval (CI) of the model, and the Youden Index was calculated to identify an optimal cut-point.

**Legend.** ROC curve of the continuous alternative ALND predictor. ROC: receiver operating characteristics; TPR: True Positive Rate; FPR: False Positive Rate.

The AUC was 0.78, (95%CI: 0.60 – 0.97) and the novel cut-point was: 60.8. The model predictor passed the Hosmer-Lemershow test for the goodness of fit with a p=1.00.

Levels superior or equal to the cut-off were significantly associated with the presence of non-SLN+ after ALND at univariate analysis (OR: 14.4, 95%CI: 2.55 – 81.3, p=0.003).

When comparing the new model with the previous one in the same casuistry with no NET-treated patients, the previous model identified 3 false positive (37.5% of all patients identified as positives) vs. 9 (56.3% of all patients identified as positive) and 4 false negatives (8.5% of all patients identified as negative) vs. 2 (5.1% of the total negatives).

# Supplementary tables

## **Supplementary table 1. Main characteristics and outcomes of the NET subpopulation**

| **DEMOGRAPHICS** | **NET Population** | | **Non-SLN-negative** | | **Non-SLN-positive** | | **χ² *P*** |
| --- | --- | --- | --- | --- | --- | --- | --- |
|  | **N** | **%** | **N** | **%** | **N** | **%** |  |
|  | 17 | 100.0 | 10 | 58.8 | 7 | 41.2 |  |
| Age at diagnosis (years) |  |  |  |  |  |  |  |
| *Mean* | 63 | - | 66 | - | 59 | - | 0.145 |
| *SD* | ±12.8 | - | ±13.7 | - | ±11.1 | - |  |
| BMI (Kg/m2) |  |  |  |  |  |  |  |
| *≥25* | 10 | 58.8 | 5 | 50.0 | 5 | 71.4 | 0.377 |
| *<25* | 7 | 41.2 | 5 | 50.0 | 2 | 28.6 |  |
| Menopause at diagnosis |  |  |  |  |  |  |  |
| Yes | 14 | 82.4 | 8 | 80.0 | 6 | 85.7 | 0.761 |
| No | 3 | 17.6 | 2 | 20.0 | 1 | 14.3 |  |
| Primary tumor size (mm) according to MRI |  |  |  |  |  |  |  |
| *Mean* | 36 | - | 37 | - | 35 | - | 0.429 |
| *SD* | 16.2 | - | ±14.1 | - | ±20.1 | - |  |
| Primary tumor size (clinical TNM category) |  |  |  |  |  |  |  |
| *cT1 (0.1 – 20.0 mm)* | 3 | 17.6 | 1 | 10.0 | 2 | 28.6 | 0.484 |
| *cT2 (20.1 – 50.0 mm)* | 10 | 58.8 | 7 | 70.0 | 3 | 42.9 |  |
| *cT3 (>50.0 mm)/cT4 (non-inflammatory)* | 4 | 23.5 | 2 | 20.0 | 2 | 28.6 |  |
| Unconfirmed suspicious cN+ at diagnosis^#^ |  |  |  |  |  |  |  |
| *Yes* | 5 | 29.4 | 2 | 20.0 | 3 | 42.9 | 0.308 |
| *No* | 12 | 70.6 | 8 | 80.0 | 4 | 57.1 |  |
| Histology type |  |  |  |  |  |  |  |
| *Ductal* | 12 | 70.6 | 8 | 80.0 | 4 | 57.1 | 0.309 |
| *Lobular/Other* | 5 | 29.4 | 2 | 20.0 | 3 | 42.9 |  |
| Tumor grade |  |  |  |  |  |  |  |
| *I* | 3 | 18.8 | 2 | 22.2 | 1 | 14.3 | 0.687 |
| *II* | 13 | 81.3 | 7 | 77.8 | 6 | 85.7 |  |
| *III* | 0 | 0.0 | 0 | 0.0 | 0 | 0.0 |  |
| *Overall* | 16 | 94.1 | 9 | 90.0 | 7 | 100.0 |  |
| Ki67% |  |  |  |  |  |  |  |
| *Mean* | 17 | - | 20 | - | 13 | - | 0.188 |
| *SD* | ±15.1 | - | ±17.4 | - | ±11.1 | - |  |
| *≤14%* | 9 | 52.9 | 4 | 40.0 | 5 | 71.4 | 0.201 |
| *>14%* | 8 | 47.1 | 6 | 60.0 | 2 | 28.6 |  |
| *Overall* | 17 | 100.0 | 10 | 100.0 | 7 | 100.0 |  |
| ER% |  |  |  |  |  |  |  |
| *Mean* | 90 | - | 89 | - | 92 | - | 0.310 |
| *SD* | ±10.2 | - | ±12.3 | - | ±6.8 | - |  |
| PR% |  |  |  |  |  |  |  |
| *Mean* | 70 | - | 65 | - | 78 | - | 0.229 |
| *SD* | ±35.5 | - | ±41.7 | - | ±25.1 | - |  |
| IHC tumor classification |  |  |  |  |  |  |  |
| *HR+/HER2-* | 17 | 100.0 | 10 | 100.0 | 7 | 100.0 | - |
| *HER2+* | 0 | 0.0 | 0 | 0.0 | 0 | 0.0 |  |
| *TN* | 0 | 0.0 | 0 | 0.0 | 0 | 0.0 |  |
| Tumor Focality |  |  |  |  |  |  |  |
| *Unifocal* | 9 | 52.9 | 6 | 60.0 | 3 | 42.9 | 0.486 |
| *Multifocal/Multicentric* | 8 | 47.1 | 4 | 40.0 | 4 | 57.1 |  |
| ET Type |  |  |  |  |  |  |  |
| *AI* | 13 | 76.5 | 8 | 80.0 | 5 | 71.4 | 0.682 |
| *Tamoxifen* | 4 | 23.5 | 2 | 20.0 | 2 | 28.6 |  |
| Breast MRI response after PST |  |  |  |  |  |  |  |
| *Progression* | 1 | 5.9 | 1 | 10.0 | 0 | 0.0 | 0.450 |
| *Partial Response ≤50% + Stable Disease* | 11 | 64.7 | 5 | 50.0 | 6 | 85.7 |  |
| *Partial Response >50%* | 4 | 23.5 | 3 | 30.0 | 1 | 14.3 |  |
| *Complete Response* | 1 | 5.9 | 1 | 10.0 | 0 | 0.0 |  |
| *Overall* | 17 | 100.0 | 10 | 100.0 | 7 | 100.0 |  |
| Type of Surgery |  |  |  |  |  |  |  |
| *Conservative* | 8 | 47.1 | 7 | 70.0 | 1 | 14.3 | **0.024** |
| *Mastectomy* | 9 | 52.9 | 3 | 30.0 | 6 | 85.7 |  |
| SLN Evaluation |  |  |  |  |  |  |  |
| *Conventional* | 5 | 29.4 | 2 | 20.0 | 3 | 42.9 | 0.309 |
| *OSNA* | 12 | 70.6 | 8 | 80.0 | 4 | 57.1 |  |
| Number of Positive SLN |  |  |  |  |  |  |  |
| *Mean* | 2 | - | 1 | - | 2 | - | **0.021** |
| *SD* | ±0.9 | - | ±0.8 | - | ±0.8 | - |  |
| Type of Positive SLN |  |  |  |  |  |  |  |
| *ITC/Micrometastasis* | 3 | 17.6 | 3 | 30.0 | 0 | 0.0 | 0.110 |
| *Macrometastasis* | 14 | 82.4 | 7 | 70.0 | 7 | 100.0 |  |
| pCR in-breast |  |  |  |  |  |  |  |
| *Yes* | 1 | 5.9 | 1 | 10.0 | 0 | 0.0 | 0.388 |
| *no* | 16 | 94.1 | 9 | 90.0 | 7 | 100.0 |  |
| Events |  |  |  |  |  |  |  |
| *Relapses* | 0 | 0.0 | 0 | 0.0 | 0 | 0.0 |  |
| *Deaths* | 0 | 0.0 | 0 | 0.0 | 0 | 0.0 | - |
| *None* | 17 | 100.0 | 10 | 100.0 | 7 | 100.0 |  |

**Legend.** SD: standard deviation; +: positive; NET: neoadjuvant endocrine therapy; ER: estrogen receptor; PR: progesterone receptor; MRI: magnetic resonance imaging; PST: primary systemic treatment; ITC: isolated tumor cells; SLN: sentinel lymph-node.

# Supplementary figures

## **Supplementary figure 1. ROC curves of the variables integrating the ALND-Predict multiparametric score showing their capability of detecting patients with non-SLN+ at ALND**

**Legend. A:** ROC curve of the PR variable; **B:** ROC curve of the sentinel lymph-node typology of affection (ITC/micrometastases vs. macrometastases) variable; **C:** ROC curve of the number of affected sentinel lymph-nodes variable; **D:** ROC curve of the Ki67 dichotomic variable. ROC: receiver operating characteristics; non-SLN+: positive/affected non-sentinel lymph-nodes; ALND: axillary lymph-nodal dissection; TPR: True Positive Rate; FPR: False Positive Rate; ITC: isolated tumor cells.
